# Pharmacokinetics, bioavailability and plasma protein binding of tolfenamic acid in rainbow trout (*Oncorhynchus mykiss*)

**DOI:** 10.1002/vms3.1533

**Published:** 2024-07-02

**Authors:** Orhan Corum, Duygu Durna Corum, Pedro Marin, Omer Faruk Acar, Mert Aksoy, Kamil Uney

**Affiliations:** ^1^ Department of Pharmacology and Toxicology, Faculty of Veterinary Medicine University of Hatay Mustafa Kemal Hatay Turkiye; ^2^ Department of Pharmacology, Faculty of Veterinary Medicine University of Murcia Murcia Spain; ^3^ Faculty of Veterinary Medicine University of Kastamonu Kastamonu Turkiye; ^4^ Department of Pharmacology and Toxicology, Faculty of Veterinary Medicine University of Selcuk Konya Turkiye

**Keywords:** bioavailability, pharmacokinetics, plasma protein binding, rainbow trout, tolfenamic acid

## Abstract

**Background:**

Although research on the mechanism and control of pain and inflammation in fish has increased in recent years, the use of analgesic drugs is limited due to the lack of pharmacological information about analgesic drugs. Tolfenamic acid is a non‐steroidal anti‐inflammatory drug and can be used in fish due to its low side effect profile and superior pharmacokinetic properties.

**Objectives:**

The pharmacokinetics, bioavailability and plasma protein binding of tolfenamic acid were investigated following single intravascular (IV), intramuscular (IM) and oral administration of 2 mg/kg in rainbow trout at 13 ± 0.5°C.

**Methods:**

The experiment was carried out on a total of 234 rainbow trout (*Oncorhynchus mykiss*). Tolfenamic acid was administered to fish via IV, IM and oral route at a dose of 2 mg/kg. Blood samples were taken at 13 different sampling times until the 72 h after drug administration. The plasma concentrations of tolfenamic acid were quantified using high pressure liquid chromatography–ultraviolet (UV) and pharmacokinetic parameters were assessed using non‐compartmental analysis.

**Results:**

The elimination half‐life (*t*_1/2ʎz_) of tolfenamic acid for IV, IM and oral routes was 3.47, 6.75 and 9.19 h, respectively. For the IV route, the volume of distribution at a steady state and total body clearance of tolfenamic acid were 0.09 L/kg and 0.03 L/h/kg, respectively. The peak plasma concentration and bioavailability for IM and oral administration were 8.82 and 1.24 µg/mL, and 78.45% and 21.48%, respectively. The mean plasma protein binding ratio of tolfenamic acid in rainbow trout was 99.48% and was not concentration dependent.

**Conclusions:**

While IM route, which exhibits both the high plasma concentration and bioavailability, can be used in rainbow trout, oral route is not recommended due to low plasma concentration and bioavailability. However, there is a need to demonstrate the pharmacodynamic activity of tolfenamic acid in rainbow trout.

## Graphical abstract

Pharmacokinetics of tolfenamic acid in rainbow trout.

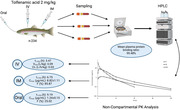

## 1 INTRODUCTION

Fish consumption has increased as people change their dietary habits to a healthy diet. Turkiye is the world's second‐largest producer of rainbow trout, following Iran (Yıldırım & Cantas, 2022). Trout is a popular inland fish species, accounting for 37.2% of our country's total fish production (Corum et al., 2023; Uney et al., 2021). Rainbow trout (*Oncorhynchus mykiss*) is economically significant due to its quick growth, tolerance of very high temperatures and appropriateness for hatchery farming (Stanković et al., 2015). In recent years, research on the mechanism and control of pain and inflammation in fish has increased. Fish have nociceptors responsible for the perception of pain, functionally similar to mammals (Greene et al., 2020). In addition, cyclooxygenase (COX) enzymes are also found in fish and are homologous to those found in mammals (Al‐Qutob & Nashashibi, 2009). Therefore, due to their analgesic and anti‐inflammatory effects, non‐steroidal anti‐inflammatory drugs (NSAIDs) can be used in adjuvant treatment of bacterial diseases, invasive procedures, traumatic injuries, tissue damage and aggression in fish (Greene et al., 2020; Martin et al., 2021). The fact that NSAIDs do not cause side effects in clinical studies in fish and that they have antipyretic and anti‐inflammatory properties provides advantages in use (Chatigny et al., 2018). In studies conducted with NSAIDs, carprofen increased feed intake in trout, ketoprofen reduced postsurgical muscle damage and required anaesthetic concentration in goldfish and ibuprofen showed an anti‐inflammatory effect in fathead minnows (Chatigny et al., 2018; Sneddon, 2012).

Tolfenamic acid, a fenamate‐derived NSAID, has analgesic, antipyretic and anti‐inflammatory effects. It works by suppressing the COX enzymes responsible for prostaglandin synthesis and it also inhibits leukotriene production (Moilanen & Kankaanranta, 1994). The action of tolfenamic acid on COX enzymes differs by animal species. While it has a non‐selective effect on COX enzymes in calves, it is especially effective on the COX‐2 enzyme in goats (Turk et al., 2021a). Tolfenamic acid is approved for use in mastitis and respiratory tract infections in cattle and goats, metritis–mastitis agalaxia infections in pigs and postoperative analgesia in cats and dogs (Anonymous, 2024; CVMP, 1997). Tolfenamic acid is used in doses of 2 and 4 mg/kg in mammals such as cattle, sheep, goats, dogs and horses (Anonymous, 2024; CVMP, 1997).

The pharmacokinetics of NSAIDs such as meloxicam (Corum et al., 2022), carprofen (Uney et al., 2021) and ketoprofen (Greene et al., 2020) were investigated in rainbow trout. Although the short elimination half‐life of meloxicam (2.95–4.55 h, Corum et al., 2022) and ketoprofen (3.91–4.40 h, Greene et al., 2020) in trout provides an advantage in acute situations, it is a disadvantage in terms of creating more stress and workload in repeated administrations. Although the elimination half‐life of carprofen in trout is long (30.66–46.11 h), it causes a decrease in activity in fish depending on the dose (Uney et al., 2021). Therefore, short‐ or long‐acting NSAIDs can be used in fish medicine according to the need, and the tolfenamic acid could be used in fish due to its low side effect profile and superior pharmacokinetic properties such as high bioavailability and long elimination half‐life (Turk et al., 2021a). However, there is no information about the use of tolfenamic acid in fish. Pharmacokinetics examines the absorption, distribution, biotransformation/metabolism and excretion processes of drugs and allows us to comment on the course and effects of the drug in the body using mathematical equations (Nishant et al., 2011). It is not possible to determine the appropriate dosage regimen without conducting pharmacokinetic studies in the target species. Therefore, pharmacokinetic studies are needed to use tolfenamic acid in fish. The aim of this study was (I) to reveal the pharmacokinetics and bioavailability of tolfenamic acid at a single dose of 2 mg/kg following intravascular (IV), intramuscular (IM) and oral administration in rainbow trout at temperatures of 13 ± 0.5°C; and (II) to determine the in vitro binding ratio of tolfenamic acid to plasma protein in rainbow trout.

## 2 MATERIALS AND METHODS

### 2.1 Chemicals

The analytical standard of tolfenamic acid (≥98%) was obtained from Sigma‐Aldrich. Acetonitrile and methanol were used in high‐performance liquid chromatography (HPLC) grade (VWR International, Fontenay‐sous‐Bois). Orthophosphoric acid was provided from Merck. Commercial preparation of tolfenamic acid (Tolfine 40 mg/mL, Novakim, Kocaeli/Turkiye) was used for drug administration to fish.

### 2.2 Animals

The experiment was carried out on a total of 234 healthy rainbow trout (215 ± 10 g of body weight) at a local fish farm (Kastamonu/Turkiye). Portion fish, the size of commercially available rainbow trout, were preferred because, due to their longevity, they are more likely to be exposed to painful and inflammatory conditions. The study included fish that had not taken any medicine within the prior 2 months and showed no symptoms of sickness or trauma. The fish were maintained in concrete ponds that were supplied with a constant supply of spring water (water temperature: 13 ± 0.5°C, pH: 8.2 ± 0.1) and exposed to natural daily lighting. The fish were placed in ponds 10 days before the study to adapt to the environment and were fed with drug‐free commercial fish feed. The experiment was approved (2022/01) by the Kastamonu University Animal Experiments Local Ethics Committee (Kastamonu/Turkiye).

### 2.3 Experimental design

For drug administration to fish, the commercial formulation of tolfenamic acid was diluted with injection water at a concentration of 2 mg/mL. A total of 234 rainbow trout were randomly divided into three equal groups: IV (*n* = 78), IM (*n* = 78) and oral (*n* = 78) according to administration route. To reduce handling and immobilisation stress, six different fish were used at each sampling time, and drug administration and blood collections were performed under anaesthesia (tricaine methanesulfonate, MS‐222, 200 mg/L). Tolfenamic acid was administered through IV (caudal vessel), IM (epaxial muscle) and oral (via the gastric gavage) routes at 2 mg/kg dose. Blood samples (2 mL) were collected from the caudal vessel by using of a 26‐G needle attached to a 2 mL syringe at the 0 (control), 0.25, 0.5, 1, 2, 4, 6, 8, 10, 12, 24, 48 and 72 h after drug administration. Following a 10‐min centrifugation at 4,000 × *g*, plasma was obtained from the blood samples and kept at −80°C until analysis.

### 2.4 Tolfenamic acid analysis

Tolfenamic acid analysis from plasma samples was performed using a HPLC–ultraviolet (UV) according to previously reported methods (Corum et al., 2019, 2018). Briefly, 100 µL of plasma was transferred to 2 mL microcentrifuge tubes. Then, 150 µL acetonitrile was added to the plasma. The mixture was vortexed for 35 s and then centrifuged at 12,000 × *g* for 12 min. The supernatant was transferred to auto‐sampler vials and 20 µL was injected into the HPLC system. HPLC system consists of a column oven (CTO‐10A), a pump (LC‐20AT), a degasser (DGU‐20A), an auto‐sampler (SIL 20A) and an UV–visible (VIS) detector (SPD‐20A). Separation was carried out with an inertsil ODS‐3 column (4.6 × 250 mm; 5 µm; GL Sciences) kept at 40°C. The UV detection wavelength was set at 289 nm. The mobile phase consisted of 0.1% orthophosphoric acid in water and acetonitrile (35:65, v/v). The flow rate was 1 mL/min.

The chromatographic procedure was validated following the guidelines provided by the European Medicines Agency (EMA, 2011). The stock solution of tolfenamic acid was prepared in acetonitrile:methanol:distilled water (4:4:2, v/v/v) to obtain a concentration of 1 mg/mL. Calibration standards (0.04–80 µg/mL) and quality control samples (0.2, 2 and 20 µg/mL) were prepared into blank fish plasma. For the purpose of determining recovery, precision and accuracy, quality control samples of tolfenamic acid at low (0.2 µg/mL), medium (2 µg/mL) and high (20 µg/mL) concentrations were utilised. The recovery was determined in six replicate by extracting quality control samples. The recovery was calculated by comparing the spiked concentrations with the observed concentration. The intra‐day and inter‐day accuracy and precision were determined by six replicate analyses of each level of quality control samples within 1 day or on 6 consecutive days. The precision was determined by the coefficient of variation (CV), and the accuracy is expressed as bias [Bias (%) = 100 × (calculated concentration − theoretical concentration)/theoretical concentration].

### 2.5 Pharmacokinetic analysis

The concentration–time data of tolfenamic acid were analysed using the WinNonlin 6.1.0.173 software (Pharsight Corporation, Scientific Consulting Inc.) through pharmacokinetic non‐compartmental analysis. Tolfenamic acid concentrations were determined for six fish at each sampling time and averaged. Pharmacokinetic data of tolfenamic acid were calculated from the average plasma concentration at sampling times, as documented in previous studies (Corum et al., 2023; Durna Corum et al., 2022). The area under the concentration (AUC) versus time curve, AUC extrapolated from *t*_last_ to ∞ in % of the total AUC (AUC_extrap_ %), volume of distribution at steady state (*V*_dss_), total body clearance (Cl_T_), terminal elimination half‐life (*t*_1/2λz_), mean residence time (MRT), mean absorption time (MAT = MRT_IM,oral_ − MRT_IV_) and bioavailability (*F* = AUC_IM,oral_ × 100/AUC_IV_) were calculated. The peak plasma concentration (*C*_max_), plasma concentration at time 0.25 h (*C*_0.25 h_) and the time to reach peak plasma concentration (*T*_max_) after tolfenamic acid administration were calculated directly from the data on the plasma concentration–time curve. AUC_IV_ and AUC_IM,oral_ were calculated using the linear/log trapezoidal method and the linear up/log down method, respectively.

### 2.6 Plasma protein binding

In plasma samples obtained from healthy fish, the plasma protein binding ratio of tolfenamic acid was evaluated using ultrafiltration method (Ural & Uney, 2021). Plasma collected from fish that were not treated with drug was pooled. Tolfenamic acid was added to plasma samples to obtain concentrations of 0.4, 4, 10 and 40 µg/mL. Three replicates were run for each concentration. It was incubated at 13°C for 30 min to ensure that the binding of tolfenamic acid to plasma proteins reached equilibrium. Afterwards, 1 mL of the sample was transferred to amicon ultra centrifugal filters (Ultracel 10 kDa, Millipore Corporation) and centrifuged at 4,000 × *g* for 15 min. The ultrafiltrate sample was analysed directly by HPLC for determination of free tolfenamic acid concentration. The plasma protein binding ratio of tolfenamic acid was determined according to the following formula: Protein binding (%) = [100 × (total drug − free drug)/total drug].

## 3 RESULTS

### 3.1 Animals

All fish remained in good health (swimming and behaviour) during the acclimatisation and experimental period.

### 3.2 Method validation

The calibration curve of tolfenamic acid was linear (*R* ^2^ > 0.9993) between 0.04 and 80 µg/mL. The recovery of tolfenamic acid ranged from 91% to 96%. The lower limit of quantification (LLOQ) was 0.04 µg/mL for tolfenamic acid in trout plasma with the CV less than 20% and the bias of ±15%. The intra‐day and inter‐day coefficients of variation were ≤5.60% and ≤6.70%, respectively. The intra‐day and inter‐day bias were ±5.3% and ±5.9%, respectively.

### 3.3 Pharmacokinetics and plasma protein binding

**FIGURE 1:**
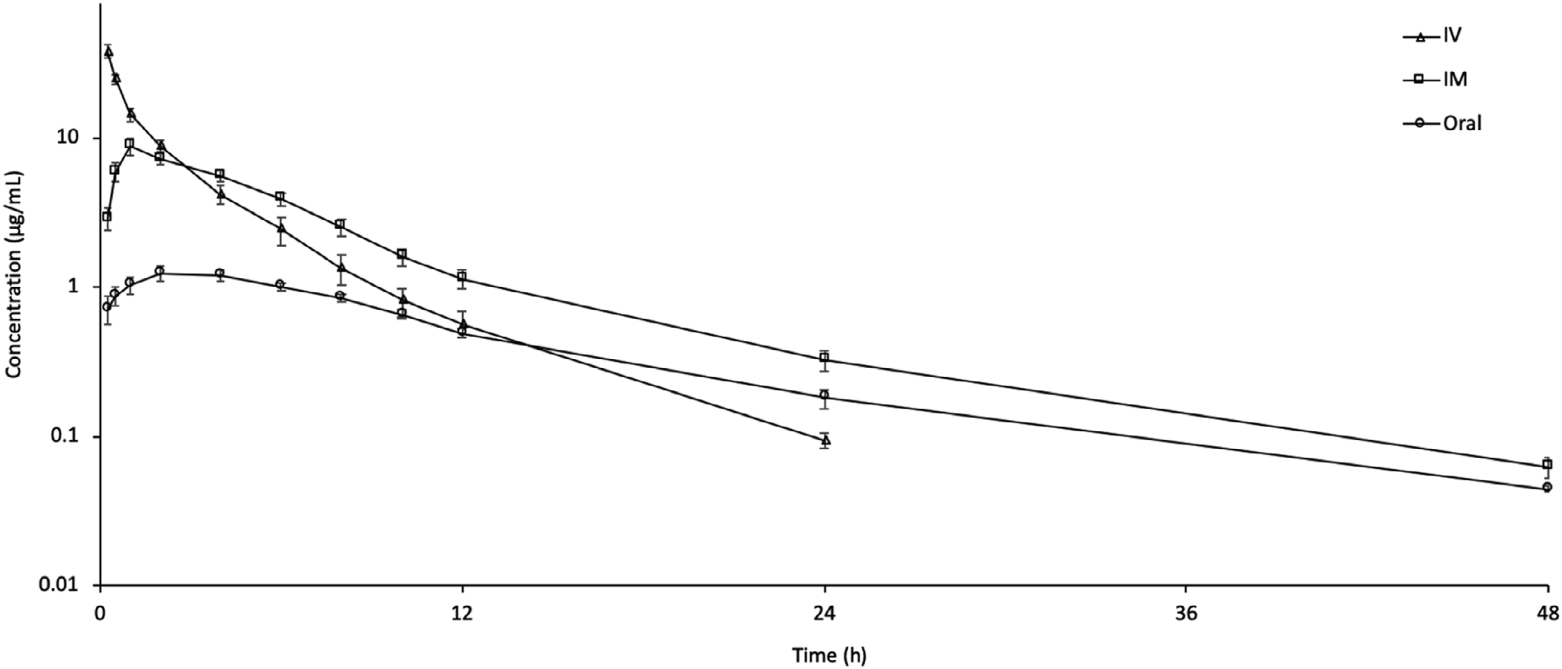
Semi‐logarithmic plasma concentration–time curves of tolfenamic acid following intravascular (IV), intramuscular (IM) and oral administrations at a single dose of 2 mg/kg in rainbow trout (*Oncorhynchus mykiss*) at 13 ± 0.5°C (*n* = 6).

Plasma concentrations of tolfenamic acid after IV, IM and oral administration at a dose of 2 mg/kg are presented in Figure 1. Tolfenamic acid was detected in plasma up to 24 h after IV administration, and 48 h after IM and oral administration. Tolfenamic concentration at the time of initial sampling point (0.25 h) was 38.34 ± 4.12, 2.88 ± 0.51 and 0.72 ± 0.15 µg/mL after IV, IM and oral administration, respectively. Plasma concentrations of tolfenamic acid dropped to 0.09 ± 0.01, 0.06 ± 0.01 and 0.04 ± 0.00 µg/mL at the final sampling times following IV, IM and oral administration, respectively. The *C*_max_ of IM and oral administration was 8.82 and 1.24 µg/mL at 1 and 2 h, respectively.

The pharmacokinetic parameters after the IV, IM and oral administrations of tolfenamic acid in rainbow trout are presented in Table 1. Tolfenamic acid exhibited a *t*_1/2ʎz_ of 3.47 h, *V*_dss_ of 0.38 L/kg and Cl_T_ of 0.03 L/h/kg after IV administration. The *t*_1/2ʎz_ and AUC values were different between administration groups. The bioavailability of tolfenamic acid was 85.87% for IM route and 25.02% for oral route. The AUC_extrap_ values for all routes were less than 20%. The plasma protein binding ratio of tolfenamic acid is presented in Table 2. The mean plasma protein binding ratio of tolfenamic acid in rainbow trout was 99.48 ± 0.14% and was not concentration dependent.

**TABLE 1:** Plasma pharmacokinetic parameters of tolfenamic acid following intravascular (IV), intramuscular (IM) and oral administrations at a single dose of 2 mg/kg in rainbow trout (*Oncorhynchus mykiss*) at 13 ± 0.5°C (*n* = 6).

Parameter	IV	IM	Oral
*t*_1/2ʎz_ (h)	3.47	6.75	9.19
AUC_0–last_ (h µg/mL)	70.18	60.07	17.09
AUC_0–∞_ (h µg/mL)	70.65	60.67	17.68
AUC_extrap_ (%)	0.67	0.99	3.31
MRT_0–∞_ (h)	3.11	8.23	13.34
MAT (h)	‐	5.12	10.23
Cl_T_ (L/h/kg)	0.03	‐	‐
*V*_dss_ (L/kg)	0.09	‐	‐
*C*_max_ (µg/mL)	‐	8.82 ± 1.11	1.24 ± 0.15
*C*_0.25 h_ (µg/mL)	38.34 ± 4.12	2.88 ± 0.51	0.72 ± 0.15
*T*_max_ (h)	‐	1.00	2.00
*F* (%)	‐	85.87	25.02

*t*_1/2λz_, terminal elimination half‐life, AUC, area under the concentration versus time curve; AUC_extrap_ %, area under the plasma concentration–time curve extrapolated from *t*_last_ to ∞ in % of the total AUC; MRT_0–∞_, mean residence time; MAT, mean absorption time; Cl_T_, total body clearance; *V*_dss_, volume of distribution at steady state; *C*_max_, peak plasma concentration; *C*_0.25 h_, plasma concentration at time 0.25 h; *T*_max_, time to reach peak plasma concentration; *F*, bioavailability.

**TABLE 2:** Plasma protein binding ratio of tolfenamic acid in rainbow trout (*Oncorhynchus mykiss*) at 13°C.

Tolfenamic acid concentration (µg/mL)	Binding ratio (%)
0.4	99.28 ± 0.16
4	99.56 ± 0.04
10	99.58 ± 0.02
40	99.50 ± 0.03
Mean ± SD	99.48 ± 0.14

## 4 DISCUSSION

In recent years, with the increase in studies on the control of pain and inflammation in fish, research on the pharmacokinetics of NSAIDs in trout has also increased (Corum et al., 2022; Greene et al., 2020; Uney et al., 2021). Tolfenamic acid can be used to control pain and inflammation in trout due to its superior pharmacokinetic properties and low side effect profile. However, since there are no previous studies in fish, the dosage regimen is adapted from other species and used extra‐label. However, depending on the anatomical and physiological differences between species, the pharmacokinetics, pharmacodynamics and toxic effects of drugs may differ. It is very important to know the pharmacokinetic data to determine the appropriate dosage regimen for the target species. In this study, the IV, IM and oral pharmacokinetics of tolfenamic acid in fish were revealed for the first time. It was determined that the pharmacokinetics of tolfenamic acid in fish varied with the administration of route and was different from other species.

In animals, parenteral and oral use of tolfenamic acid at a doses of 2–4 mg/kg is recommended (CVMP, 1997). Therefore, tolfenamic acid was administered to rainbow trout at a dose of 2 mg/kg. No adverse effects were observed after IV, IM and oral administration of tolfenamic acid to rainbow trout at a dose of 2 mg/kg. The side effect of tolfenamic acid is generally low and well tolerated in mammals, birds and reptiles at doses of 2–4 mg/kg (Corum et al., 2024; Raweewan et al., 2020a, 2020b; Yildiz et al., 2019).

The tolfenamic acid after IV administration in rainbow trout exhibited short *t*_1/2ʎz_ with 3.47 h, which was similar to that previously reported in mammals (1.57–6.09 h, Jaussaud et al., 1992; Tekeli et al., 2020) and birds (1.51–1.95 h, Cetin et al., 2022; Turk et al., 2021b), it was shorter than reported in turtles (17.55–38.92 h, Corum et al., 2019; Raweewan et al., 2020b). Fish are heterotherms and their metabolic rates are approximately 10 times lower than mammals (Van de Pol et al., 2017). In addition, the metabolic rate of the fish is affected by the water temperature, and the metabolic rate decreases at low water temperature (Corum et al., 2023). Therefore, in this study conducted at low water temperature, while we expected the *t*_1/2ʎz_ of tolfenamic acid to be longer than mammals, it was a surprise for us that it was similar to mammals. Similarly, the *t*_1/2ʎz_ of NSAIDs such as meloxicam and carprofen in rainbow trout was also found to be shorter than expected (Corum et al., 2022; Uney et al., 2021). As *t*_1/2ʎz_ is a hybrid parameter (*V*_d_/Cl_T_), the variability of *t*_1/2ʎz_ between species may result from the difference in *V*_d_ and Cl_T_ (Toutain & Bousquetmélou, 2004).

The *V*_dss_ of following IV administration of tolfenamic acid in rainbow trout was 0.09 L/kg, which was smaller than that previously reported in mammals (0.37–1.1 L/kg, Lefebvre et al., 1997; Turk et al., 2021a), birds (0.25–0.41 L/kg, Cetin et al., 2022; Turk et al., 2021b) and red‐eared slider turtles (0.30 L/kg, Corum et al., 2019), and larger than that previously reported in green sea turtles and Hawksbill turtles (0.03–0.05 L/kg, Raweewan et al., 2020a, 2020b). Body composition and the binding ratio of drugs to plasma proteins influence *V*_dss_ (Martinez & Modric, 2010). In general, the *V*_dss_ of drugs that are highly bound to plasma proteins are low. In this study, the plasma protein binding ratio of tolfenamic acid was 99.5%. The binding ratio of tolfenamic acid to plasma proteins was 99.7% for human (Pentikäinen et al., 1981), 99% for dogs (Lefebvre et al., 1997), 91%–94% for calves (Dinakaran & Dumka, 2013) and 19%–31% for turtles (Raweewan et al., 2020a; 2020b). The total protein content of trout plasma is lower than human plasma, and rainbow trout contains two albumin‐like proteins that are not identical structure to human albumin (Henneberger et al., 2022). The difference in the binding ratio of tolfenamic acid to plasma proteins between species may be due to differences in albumin affinity and binding sites. Breast cancer resistance protein (BCRP) is an important transport protein in the ABC transporter family, and since it has an efflux character, it acts as a pump that takes its substrate drugs out of the cell. Tolfenamic acid is a BCRP substrate and its tissue accumulation is affected by BCRP activity (Blanco‐Paniagua et al., 2021). Although BCRP transport protein is found in animals, including fish, its activities vary among animal species (Li et al., 2008; Zaja et al., 2016). The variability of *V*_dss_ of tolfenamic acid among animal species may be due to differences in plasma protein binding ratio, BCRP activity and body composition.

The Cl_T_ of following IV administration of tolfenamic acid in rainbow trout was 0.03 L/h/kg, which was higher than that previously reported in turtles (0.001–0.01 L/h/kg, Corum et al., 2019; Raweewan et al., 2020a, 2020b), and lower than that that previously reported in mammals (0.07–0.30 L/h/kg, Jaussaud et al., 1992; Tekeli et al., 2020) and birds (0.15–0.16 L/h/kg, Cetin et al., 2022; Turk et al., 2021b). Tolfenamic acid undergoes significant hepatic metabolism and is eliminated in the urine and bile alongside its metabolites. The metabolism of tolfenamic acid varies significantly between species, with the fraction excreted unchanged in urine ranging from 1% to 89% (CVMP, 1997; Kuninaka et al., 1981). There is no information on the metabolism and excretion of tolfenamic acid in fish. Fish have enzymes that play a role in the biotransformation of drugs, and the kidney, bile and gill play a role in their excretion (Christiansen et al., 1996; Evans et al., 2005). However, the enzyme activity and metabolic rate in fish are lower than in mammals (Kleinow et al., 1992). Similarly, the metabolic rate of turtles, which are heterotherms, is one‐seventh of that of mammals (Raweewan et al., 2020a). The reason why the Cl_T_ of tolfenamic acid in fish is lower than in mammals and birds may be differences in metabolic and excretion pathways.

The *C*_max_ of tolfenamic acid at 2 mg/kg dose in rainbow trout was 8.82 ± 1.11 µg/mL at 1 h for the IM route and 1.24 ± 0.15 µg/mL at 2 h for the oral route. Similar results have been previously reported in mammals and birds (Corum et al., 2018; Turk et al., 2021c, 2021a). These results show that the absorption extent of tolfenamic acid varies depending on the route of administration. *C*_max_ consists of the absorption extent, Cl_T_ and *V*_dss_ of the drug, and the change in these parameters affects *C*_max_ (Turk et al., 2021b). The bioavailability of tolfenamic acid after IM and oral administration in rainbow trout was 85.87% and 25.02%, respectively. The high bioavailability of tolfenamic acid after IM administration has also been previously reported in mammals (64.46%–163%), reptiles (72.02%–110.28%, Corum et al., 2019; Raweewan et al., 2020a) and birds (87.91%, Turk et al., 2021b). Oral bioavailability in rainbow trout is similar to that previously reported in goats (19.46%, Turk et al., 2021a), and lower than that reported in geese (76%, Turk et al., 2021b) and sheep (107%, Corum et al., 2018). Low oral bioavailability indicates that the drug is not well absorbed from the gastrointestinal tract. Tolfenamic acid binds to food contents in the stomach (Landoni et al., 1996) which reduces the oral absorption of tolfenamic acid and results in low bioavailability.

The *t*_1/2ʎz_ obtained after IM (6.75 h) and oral (9.19 h) administration of tolfenamic acid to rainbow trout was longer than that after IV (3.47 h) administration. Compared to IV administration, *t*_1/2ʎz_ has been reported to be longer in mammals (Corum et al., 2018; Turk et al., 2021a), birds (Turk et al., 2021b) and turtles (Corum et al., 2019; Raweewan et al., 2020a, 2020b) after extravascular administration. It has been stated that the longer *t*_1/2ʎz_ of tolfenamic acid after extravascular administration is due to the flip‐flop phenomenon (Corum et al., 2018; Lees et al., 1998; Turk et al., 2021a). The flip‐flop phenomenon occurs when the absorption rate of the drug is slower than the elimination rate (Yáñez et al., 2011). In this case, the MAT must be longer than the MRT obtained after IV administration (Yáñez et al., 2011). In rainbow trout, the MAT values obtained for extravascular routes were longer than the MRT values obtained for the IV route. In this study, the reason for the long *t*_1/2ʎz_ after extravascular administration may be the flip‐flop phenomenon.

There are no pharmacodynamic studies on the analgesic and anti‐inflammatory activity of tolfenamic acid in fish. The IC_50_ value of tolfenamic acid for exudate PGE_2_ and plasma TxB_2_ in mammals was 0.07–0.23 and 0.26–1.3 µg/mL, respectively (Lees et al., 1998; McKellar et al., 1994; Sidhu et al., 2006). While the required IC_50_ value for inhibition of PGE_2_ and TxB_2_ was obtained after IM injection at a dose of 2 mg/kg, the required IC_50_ value for inhibition TxB_2_ could not be reached after oral administration. Although tolfenamic acid remains in the exudate longer than in plasma, its exudate concentration is lower than the plasma concentration (Sidhu et al., 2005, 2006). Therefore, there is a need to demonstrate the analgesic and anti‐inflammatory activity of tolfenamic acid in fish.

If the pharmacokinetic parameters obtained with tolfenamic acid are compared with other NSAIDs studied in rainbow trout, it can be observed that the *t*_1/2ʎz_ (3.47 h) obtained for IV administration was similar to that previously reported for meloxicam (3.63 h, Corum et al., 2022) and ketoprofen (3.91 h, Greene et al., 2020), but was shorter than that reported for carprofen (30.66 h, Uney et al., 2021). The IM bioavailability (85.87%) of tolfenamic acid in rainbow trout was high as previously reported for meloxicam (78%, Corum et al., 2022), ketoprofen (85%, Greene et al., 2020) and carprofen (122%, Uney et al., 2021). However, the oral bioavailability of tolfenamic acid was low (25.02%), as previously determined for meloxicam (21%, Corum et al., 2022) and carprofen (41%, Uney et al., 2021). IM route of tolfenamic acid in fish is more appropriate since it has high bioavailability. However, considering that the most preferred route of administration in fish is oral, new oral formulations with higher bioavailability are needed.

In this study, drug administration and blood collection from rainbow trout were performed under MS‐222 anaesthesia. Although anaesthesia is effective in reducing handling and immobilisation stress (Wagner et al., 2003), it may affect the body distribution of the drug. MS‐222 anaesthesia did not affect the pharmacokinetics of florfenicol in Nile tilapia (Rairat et al., 2021). However, there is no information about the effect of MS‐222 anaesthesia on the pharmacokinetics of tolfenamic acid. Therefore, it should be noted that this research on rainbow trout was carried out under MS‐222 anaesthesia.

## 5 CONCLUSION

Tolfenamic acid after oral and IM administration remained in the body during 48 h in trout. While bioavailability after IM injection was good (86%), it was low (25%) after oral route. These data show that there is a need to develop formulations with high bioavailability or to high doses than 2 mg/kg for oral use as IM route is not a widely preferred route in fish. Moreover, this NSAID reached high peak plasma concentrations within a short period of time (*T*_max_ = 1 h) after IM administration, therefore, it could be an effective analgesic and anti‐inflammatory drug in rainbow trout. However, further efficacy, pharmacodynamic and multiple‐dose studies with tolfenamic acid are needed in trout.

## AUTHOR CONTRIBUTIONS

**Orhan Corum**: Conceptualisation; Investigation; Methodology; Project administration; Resources; supervision; Writing – original draft; Writing – review & editing. **Kamil Uney**: Conceptualisation; Investigation; Methodology; Project administration; Resources; supervision; Writing – original draft; Writing – review & editing. **Duygu Durna Corum**: Conceptualisation; Investigation; Methodology; Project administration; Resources; supervision; Writing – original draft; Writing – review & editing. **Omer Faruk Acar**: Investigation; Methodology. **Mert Aksoy**: Investigation; Methodology. **Pedro Marin**: Writing – original draft; Writing – review & editing.

## CONFLICT OF INTEREST STATEMENT

The authors declare no conflicts of interest.

### ETHICS STATEMENT

The experiment was approved (2022/01) by the Kastamonu University Animal Experiments Local Ethics Committee (Kastamonu, Turkiye), and carried out in accordance with the European Directive (2010/63/EU).

### PEER REVIEW

The peer review history for this article is available at https://www.webofscience.com/api/gateway/wos/peer-review/10.1002/vms3.1533.

## Data Availability

The data that support the findings of this study are available from the corresponding author upon reasonable request.
